# What is driving the relationship between height and cognition? Evidence from the Twins Early Development Study

**DOI:** 10.1016/j.ehb.2022.101174

**Published:** 2022-08-17

**Authors:** Vikesh Amin, Jason M. Fletcher

**Affiliations:** a Department of Economics, Central Michigan University, United States; b La Follette School of Public Affairs, University of Wisconsin-Madison, United States; c IZA, United States; d NBER, United States

**Keywords:** I1, Height, Cognition, Twins, Polygenic Scores

## Abstract

Taller children tend to have better cognitive ability, and the relationship between height and cognition has been proposed as an explanation for the height-wage labor market premium. Height-cognition associations may arise due to social factors that favor taller individuals or be driven by “common factors” that are correlated with height and cognition. Indeed, there is now evidence of a genetic correlation between height and cognition that provides specific evidence for this concern. We examine whether genetic factors explain the relationship by estimating associations between childhood height and cognition in the Twins Early Development Study. We find that height is associated with better cognition even after controlling for genetic and environmental factors shared by twins. The association between height and cognition within fraternal twin pairs is also robust to controlling for individual genetic predictors of height and cognition. These results suggest that genetic factors are not solely responsible for driving the relationship between height and cognition.

## 1. Introduction

A small number of studies have documented robust positive associations between height in childhood and adolescence with cognition. Case and Paxson (2008) found that a one standard deviation increase in height at age 7 was associated with a 0.10 standard deviation increase in reading test scores at age 7 and verbal language test scores at age 11 in the National Child Development Study (NCDS, a cohort of children born in the 3rd week of March 1958 in the UK). Using the 1979 National Longitudinal Study of Youth (NLSY79) child and young adult surveys, they also found that a one standard deviation increase in height at ages 5–6 was associated with 0.05 standard deviation increase in PIAT math and reading comprehension test scores. Using the Avon Longitudinal Study of Parents and Children (ALSPAC, a cohort of children born between 1 April 1991 and 31 December 1992 in Avon, England) von Hinke Kessler Scholder et al. (2013) found that a one standard deviation increase in height at age 8 was associated with a 0.06 standard deviation increase in IQ test scores at age 8. Understanding the origins of the height-cognition relationship is important because height is associated with economic outcomes. Taller individuals have higher labor market earnings (Case and Paxson, 2008, 2010; Böckerman and Vainiomäki, 2013, 2014, 2014; Persico et al., 2004, 2015, 2015; Thomas and Strauss, 1997), which has been proposed to arise because “height is positively related to cognitive ability, which is rewarded in the labor market” (Case and Paxson, 2008 p.499).^1^ Height and cognition are also associated with better health and lower mortality (Calvin et al., 2011; Khetan et al., 2021; Koch et al., 2011).

Two explanations have been proposed for the relationship between childhood/adolescent height and cognition. The relationship could be driven by social factors. Height is a socially desirable trait that is linked to positive attributes such as intelligence, competence, attractiveness and social status, which leads to more favorable treatment that has beneficial consequences. For example, teacher perceptions of a child’s ability may affect academic development by influencing achievement goals that teachers set. Taller children could have greater self-esteem because they are treated differently by teachers and perform better on tests. This type of discrimination in the education system may suggest an accumulation of the effects of height as children grow older, which eventually becomes evident in the height-wage premium and in height-education disparities.^2^

Alternatively, the relationship between height and cognition may be driven by unobserved common factors that are correlated with height and cognition. One example is the pre and postnatal environment that links nutrition to height and cognitive development. Another possibility is genetics. There is a positive genetic correlation of about 0.1 between height and cognition (Sniekers et al., 2017), indicating that genes associated with increased height are also associated with increased cognition, through pleiotropic effects. As an example, Sniekers et al. (2017) found that the rs2490272 (6q21) gene in an intronic region of FOXO3 has the strongest association with intelligence. This gene is part of the insulin/insulin-like growth factor 1 signaling pathway, which is associated with height. Shared family-level factors such as parental care could also generate associations between height and cognition. The direction of causation could also go in the opposite direction with cognition affecting height. For example, parents may make greater nutritional investments in the child who is perceived to be smarter leading to height gains, or children may change their eating habits after performing poorly on cognitive tests affecting their height growth.

Only three studies have attempted to estimate childhood height-cognition associations controlling for unobserved factors. In the NLSY79 child and young adults survey, Case and Paxson (2008) found that the association between height at ages 5–6 and PIAT math and reading comprehension test scores was reduced from 0.05 to 0.03 standard deviations after controlling for genetic and family factors shared by siblings through the inclusion of mother fixed effects. Using the same dataset Case and Paxson (2010) found positive associations between height and cognition at ages 10–11 in regressions models with mother fixed effects. Case and Paxson (2008, 2010) though cannot rule out genetics as an explanation for the height-cognition relationship, because sibling share 50% of their genes on average. Social factors also cannot be ruled out because taller siblings could be treated more favorably by parents or teachers. von Hinke Kessler Scholder et al. (2013) used a genetic score based on nine single nuclear polymorphisms (SNPs) as an IV to estimate associations between childhood height and IQ at age 8 in the ALSPAC cohort. They found statistically significant associations of height with IQ for girls, but not for boys. Interpreting their findings as causal relies on assuming (amongst others) that height-increasing SNPs only affect cognition through their effect on height. This could be violated because genes associated with height may be related to other genes or traits that affect cognition. Therefore, it remains unclear in the broader literature whether height is associated with cognition because of social or common factors.

We contribute by examining the role of common factors, particularly genetics, as an explanation for the height-cognition relationship. We estimate associations between childhood height and cognition in the Twins Early Development Study (TEDS) using both types of twin comparisons, MZ (monozygotic; identical) and DZ (dizygotic, fraternal). MZ twins are genetically identical so that MZ-differences control for genetics. DZ twins, like non-twin siblings, share 50% of their genes on average. A useful aspect of TEDS is that it contains polygenic scores (PGSs) for height and educational attainment. A PGS is a summary measure of genetic predisposition, which aggregates the effects of SNPs that are found to be associated with a trait in genome-wide association studies (GWAS).^3^ The combination of DZ-differences and PGS controls can mimic the MZ-difference design. This paper thus represents the only work that "fully" accounts for genetics with MZ twins and also provides an extension of sibling fixed effects with additional controls for genetics that vary between siblings, which are both innovations in the literature. We find that height is associated with better cognition within MZ twin pairs and DZ twin pairs, controlling for education and height PGSs. Our results suggest that genetic factors are unlikely to be driving the relationship between height and cognition.

## 2. Data

TEDS is a longitudinal study of twins born in England and Wales between 1994 and 1996. Twin births between January 1994 and December 1996 were identified through birth records. The UK Office for National Statistics contacted these families after screening for infant mortality, and 16,810 families responded to acknowledge their interest in taking part in the study. The TEDS team made their first contact with these families when the twins were about 18 months of age to collect general demographic information, including zygosity and information about pregnancy and birth. Since enrollment families have been invited to take part in studies when twins were aged 2, 3, 4, 7, 8, 9, 10, 12, 14, 16, 18 and 21 years. DNA samples were obtained from 12,500 twins and genotyped on the Affymetrix GeneChip 6.0 or the Illumina HumanOmniExpressExome chips. After stringent quality control, genotyped data is available for 3706 unpaired twins (mostly MZ, but also some DZ) and 3320 DZ twin pairs. Both the overall TEDS sample and the genotyped subsample have been shown be to representative of families in the UK with small children born in the late 1990s and early 2000s (Haworth et al., 2013; Rimfeld et al., 2019; Selzam et al., 2017). .^4^

We have data on height and cognition at age 7, 12 and 16, but we restrict our analysis to age 7. This is because the number of families invited to participate, and response rates differ by survey year and instrument. Supplementary Table S1 provides information on the number of parents and twin pairs contacted and response rates at 1st contact and ages 7, 12, 16. At age 7, 14,581 parents were contacted but only 7909 parental questionnaires were returned. TEDS does not follow all twins longitudinally either. For example, there were more parents contacted at age 16 than at age 12. For cognitive testing at age 7, 9811 families were contacted of which 5727 consented and there are 5533 twin pairs where both twins completed the tests. These differences in response rates, and different families/twins being contacted leads to differences in sample composition that make comparisons across ages difficult. We nevertheless report results for contemporaneous associations between height and cognition at ages 12 and 16 in Supplementary materials.

Height was reported by parents at age 7, and twins undertook two verbal (the Similarities and the Vocabulary subtests from the WISC-III-UK) and two nonverbal (the Picture Completion subtest from the WISC-III-UK and the Conceptual Grouping subtest from the McCarthy Scales of Children’s Abilities) tests, which were administered via telephone. All test scores were standardized, and cognition is measured as the average of the standardized test scores. We have data on 11,197 twins for whom parents reported height. We drop 4047 twins with no cognition data and 515 twins with missing data on covariates. The covariates are gender, age in months, ethnicity, birthweight, and family SES.^5^ We then drop 89 unpaired twins, leaving an analytical sample of 6546 twins (4160 DZ of which 2498 are genotyped and 2386 MZ).

Summary statistics for the analytical sample are shown in Table 1. The average height of DZs is about 122.75 cm and the average cognition score is 0.14 (column 1). Summary statistics for the sub-sample of genotyped DZ twins (column 2) are similar to those for the full sample of DZ twins. There are however some notable differences in the summary statistics for MZ twins. First, the average height of MZ twins is slight lower (122.13 cm) and MZ twins have a substantially lower mean cognition score (0.03) compared to DZ and genotyped DZ twins. MZ twins have a lower birthweight on average and come from lower SES families. The average childhood SES index for MZ twins is 0.07 whereas it is 0.024 for DZ twins.^6^ Columns 4 and 5 indicates differences in height, cognition, childhood SES and birthweight between MZ and DZ/genotyped DZ twin pairs are all statistically significant. There is also a fair amount of within-twin pair variation in height and cognition. The absolute within DZ (MZ) twin pair difference in height is 4.70 (1.67) cm and in cognition is 0.76 (0.62).

## 3. Empirical framework

Our analysis was preregistered at the Open Science Framework (https://osf.io/av7wr). We first estimate OLS regressions (Eq. 1) where the cognition score of twin i in pair j (*Cognition_ij_*) is regressed on the height of twin i in pair j (*Height_ij_*) and the covariates (age in months, gender, ethnicity, family SES, and birthweight).^7^

(1) $$Cognition_{ij}=\beta_{0}+\beta Height_{ij}+\;\;X_{ij}^{\prime }\delta +u_{ij}$$

OLS estimates though are likely to be biased because of unobserved genetic and family factors that affect height and cognition. To control for some of these factors, we estimate twins fixed effect regressions (Eq. 2), where within-twin pair differences in cognition are related to within-twin pair differences in height and birthweight.


(2) $$\Delta Cognition_{j}=\beta_{0}+\beta \Delta Height_{j}+\;\;\gamma \Delta Birthweight_{j}\;\;+\Delta u_{j}$$

Twin comparisons controls for environmental factors shared by twins. Genetic factors are also fully controlled for within MZ twin pairs, but only partially within DZ twin pairs, who like ordinary siblings share 50% of their genetic makeup on average. For genotyped DZ twins we also estimate Eq. (3), which controls for differences in measured education (*ΔPGS_Edu_j_*) and height genetics (*ΔPGS_Height_j_*) to mimic the MZ twin differences design.^8^

(3) $$\Delta Cognition_{j}=\beta_{0}+\beta \Delta Height_{j}+\theta_{1}\Delta PGS\_Edu_{j}+\theta_{2}\Delta PGS\_Height_{j}+\;\;\gamma \Delta Birthweight_{j}\;\;+\Delta u_{j}$$

## 4. Results

Results are presented in Table 2. In all the analyses, the cognition score was z-standardized, and height was adjusted for age and sex using the British 1990 growth charts and then z-standardized. OLS estimates in column 1 for DZ twins shows that a one standard deviation increase in height is associated with a 0.09 standard deviation increase in cognition. This is in line with OLS estimates from the NCDS and ALSPAC cohorts reported in Case and Paxson (2008) and von Hinke Kessler Scholder et al. (2013). Controlling for unmeasured environmental and genetic factors through twin comparisons in column 2 reduces the estimated association by half to 0.04 but remains statistically significant at the 10% level. Results for genotyped DZ twins are similar. After controlling for common genetic and environmental factors in column 4, a one standard deviation increase in height is associated with a 0.05 standard deviation increase in cognition. The point estimate however is statistically insignificant likely due to higher imprecision from a smaller sample size. Controlling for measured height and education genetics in column 5 does not attenuate this association, but rather it increases to 0.06. We find no evidence that genetic factors related to height affect cognition after controlling for actual height. The coefficient on the height PGS is negative (−0.01) and statistically insignificant. In contrast, a one standard deviation in the education PGS is associated with a 0.12 standard deviation increase in cognition.

Results for MZ twins are in columns 6 & 7. The OLS estimate indicates that a one standard deviation increase in height is associated with a 0.05 standard deviation increase in cognition. This association is smaller than the OLS associations for DZ twins likely due to differences in sample composition. After controlling for shared environmental and genetic factors, the association doubles to 0.10. Why does the association increase after controlling for shared genetic and environmental factors? As MZ twins are genetically identical, any height variation must reflect differences in environmental exposures. One possibility is that parents reallocate resources to the taller twin, leading to larger height and cognition differences. The results could also be explained by environmental shocks. For example, Hwang et al. (2013) found that childhood infections were associated with height differences in 140 MZ twin pairs from the California Twin Program, independent of birth length and diet. Childhood infections may also have detrimental effects on childhood schooling/cognition leading to larger within twin pair height and cognition differences.

Supplementary materials provide results from two other analyses. First, we examined gender differences in height-cognition associations by interacting height with a dummy variable for being female in twins fixed effect regressions (Table S2). We cannot make strong inferences about gender differences due to a lack of power—estimates for height, the female dummy and the interaction between height and female are all statistically insignificant. The interaction between height and female for MZ twin pairs is large in magnitude (0.15) indicating stronger associations of height with cognition for women but is imprecisely estimated. Second, we estimated height-cognition associations at ages 12 and 16 (Table S3).^9^ We found a one standard deviation increase in height is associated with about a 0.05 standard deviation increase in cognition at age 12, within DZ, genotyped DZ and MZ twin pairs. At age 16 there are no statistically significant associations between height and cognition within DZ and genotyped DZ twin pairs though the associations are positive, whereas the within-MZ twins association is 0.10 of a standard deviation.

## 5. Summary

The relationship between height and cognition has been proposed as an explanation for the height-wage labor market premium. While there are robust associations between height and cognition, it remains unclear what mechanism is behind the associations. The associations may be due to social factors such as discrimination/perceptions where taller children are treated more favorably. Alternatively, they may be driven by unobserved common factors such as genetics, pre and postnatal environments, or family-level factors correlated with height and cognition. We examine the role of genetic factors as an explanation, by estimating the effect of childhood height on cognition using twins fixed effect models with data from TEDS. Twins fixed effects improves upon previous estimates with sibling data as it controls for more shared unobserved factors. We are also able to control for some of the unobserved genetic heterogeneity between DZ twins, by including height and education PGSs in our fixed effects regressions. We find that height-cognition associations are robust to controlling for shared genetic and environmental factors by both DZ and MZ twins. The height-cognition association within DZ twin pairs does not substantially change after controlling measured height and education genetics. This suggests that the underlying relationship between height and cognition is unlikely to be due to genetics. The effect of height on cognition could run through discrimination/perceptions or environmental shocks, which we cannot rule out.

## Supplementary Material

Supplementary Material

## Figures and Tables

**Table 1:** Summary statistics.

Sample	DZ Twin Pairs	Genotyped DZ Twin Pairs	MZ Twin Pairs	DZ– MZ Twin Pairs	Genotyped DZ–MZ Twin Pairs
	(1)	(2)	(3)	(4)	(5)
Height (cm)	122.75 (7.67)	122.85 (7.29)	122.13 (7.14)	0.62**	0.72***
Cognition	0.14 (0.97)	0.17 (0.96)	0.03 (0.97)	0.11***	0.14***
Female	0.50 (0.50)	0.51 (0.50)	0.53 (0.50)	−0.03**	−0.02*
White	0.95 (0.21)	1.00 (0.00)	0.96 (0.20)	0.01	0.04***
Childhood SES	0.24 (0.97)	0.25 (0.96)	0.07 (0.96)	0.17***	0.18***
Birth weight (grams)	2563 (534)	2565 (522)	2463 (539)	100***	102***
*Absolute within-twin pair difference* in					
Height (cm)	4.70 (4.83)	4.63 (4.68)	1.67 (2.86)	3.03***	2.96***
Cognition	0.76 (0.61)	0.76 (0.61)	0.62 (0.48)	0.15***	0.15***
Birth weight (grams)	325 (285)	320 (281)	301 (271)	24**	19***
N	4160	2498	2386		

*Notes:* Standard deviation in parentheses.

*** p < .01

** p < .05 p < .10

**Table 2:** Association between height and cognition at age 7.

Sample	DZ Twin Pairs	DZ Twin Pairs	Genotyped DZ Twin Pairs	Genotyped DZ Twin Pairs	Genotyped DZ Twin Pairs	MZ Twin Pairs	MZ Twin Pairs
Method	OLS	FE	OLS	FE	FE	OLS	FE
	(1)	(2)	(3)	(4)	(5)	(6)	(7)
Std Height	0.0868*** (.0172)	0.0433* (.0250)	0.0836*** (.0216)	0.0520 (.0320)	0.0574* (.0334)	0.0463* (.0235)	0.0981** (.0464)
Std Height PGS					−0.0103 (.0332)		
Std Education PGS					0.1248*** (.0326)		
R^2^	0.094	0.002	0.092	0.008	0.021	0.109	0.008
N	4160	4160	2498	2498	2498	2386	2386

*Notes:* OLS regressions control for age, gender, birthweight, family SES, and ethnicity. Cognition is standardized to have a mean of 0 and standard deviation of 1. Standard errors clustered at the twin pair level.

*** p < .001

** p < .05

* p < .10

Std: Standardized. The within R^2^ from twins fixed effect regressions is reported in columns 2, 4,5, and 7.

## Footnotes

1 Case and Paxson (2008) found that the height-wage premium was substantially reduced after controlling for childhood test scores. For example, in the NCDS the inclusion of childhood test scores reduced the height premium in average hourly earnings by 48% for men and 63% for women.

2 Some studies have also shown that childhood/adolescent height is associated with better academic performance and higher adult educational attainment. Cinnirella et al. (2011) found that a one-centimeter increase in height was associated with a 1.6% point increase in the probability of attending Gymnasium (the most academic secondary school track in Germany) for boys. Gorry (2017) found that an extra inch of height in adolescence was associated with 0.05 additional years of education in the US National Longitudinal Study of Adolescent to Adult Health.

3 Specifically, the PGS for individual *i* is a weighted average across the number of SNPs (m) of the number of reference alleles A (0, 1 or 2) at that SNP multiplied by the corresponding beta estimate from the GWAS analysis: $PGS_{i}=\sum_{j=1}^{m}\beta_{j}\;\;A_{ij}$.

4 For example, at first contact 44.8% of fathers had A-levels or higher and the corresponding national equivalent for families with small children born in the late 1990s and early 2000s was 47% (see Table 1 in Rimfeld et al., 2019).

5 Family SES is based on measures of maternal and paternal qualifications and occupations, and maternal age at first childbirth, which were standardized and summed.

6 Jelenkovic et al. (2015) combine data from 54 twin cohorts and document that DZ twins are considerably taller than MZ twins in childhood, with differences up to 2 cm. They discuss explanations related to lower birthweight due to intrauterine growth restriction, and that DZ twin parents might be different from MZ twin parents. They note that (i) mothers of DZ twins are significantly taller, heavier and more likely to smoke than mothers of MZ twins, and (ii) mothers of DZ twins conceived via fertility treatments are more educated and financially better off than mothers who conceived twins naturally. These explanations could also explain differences in cognition and SES observed in Table 1.

7 One possibility to deal with reverse causation is to use lagged height in Eq. (1), which has been done by other researchers (e.g., Case and Paxson, 2008). However, this does not fully resolve the issue because lagged height is highly correlated with contemporaneous height. In our data, the correlation between height at age 7 and age 12 for MZ twins the correlation is 0.59 and 0.56 for DZ twins. Reverse causality may also not be a large problem. We estimated OLS associations between cognition at age 7 and height growth between ages 7–12 controlling for gender, ethnicity, birthweight, family SES and zygosity. We found that a one standard deviation increase in cognition at age 7 was associated with a 0.10 cm increase in height between ages 7–12. This estimate was statistically insignificant and represents a small effect of 0.38% relative to the mean increase in height between ages 7–12 (26.45 cm).

8 The height and education PGSs are based on GWAS studies by Yengo et al. (2018) and Lee et al. (2018) respectively. The PGSs are not adjusted for the genotyping chip used and population stratification (the non-random distribution of genes across population subgroups). We account for this by regressing the first ten principal components of the genetic data and a dummy variable indicating the chip used for genotyping on the PGSs, and subsequently use the z-standardized residuals in the regression analysis.

9 Section B in Supplementary material provides information on height and cognition measures at age 12 and 16. Supplementary Table S4 provides summary statistics for the analytical samples in Table S3.
