# Supplementary material for: What is driving the relationship between height and cognition? Evidence from the Twins Early Development Study
Source: Econ Hum Biol. Author manuscript; Available in PMC 2023 Jan 24. (PMC9872705; doi:10.1016/j.ehb.2022.101174)
Supplement: Supplementary Material [file NIHMS1865964-supplement-Supplementary_Material.docx]

**SUPPLEMENTARY MATERIALS**

**SECTION A: ADDITIONAL TABLES**

**Table S1: TDS Sample Size by Study Year and Survey Instrument**

| **Study Year** | **Parent Questionnaire** | | **Twins Questionnaire** | | **Cognitive Testing** | | |
| --- | --- | --- | --- | --- | --- | --- | --- |
|  | **No. of parents Contacted** | **No. of parent returns** | **No. of twin pairs contacted** | **No. of twin pairs where both twins completed survey** | **No. of families contacted** | **No. of giving parental consent for testing** | **No. of twin pairs where both twins completed all tests** |
| 1^st^ Contact | 16,302 | 13,488 |  |  |  |  |  |
| Year 7 | 14,581 | 7,909 |  |  | 9,811 | 5,727 | 5,533 |
| Year 12 | 8,438 | 5,854 | 8,438 | 5,883 | 8,438 | 5,899 | 3,993 |
| Year 16 | 10,874 | 5,123 | 10,784 | 5,078 | 6,281 | 3,279 | 2,214 |

**Table S2: Gender Differences in Height-Cognition Associations at Age 7**

| **Sample** | **DZ Twins Pairs** | **Genotyped DZ Twin Pairs** | **MZ Twin Pairs** |
| --- | --- | --- | --- |
| **Method** | **FE** | **FE** | **FE** |
|  | **(1)** | **(2)** | **(3)** |
| Std Height | 0.0404  (.0291) | 0.0438  (.0410) | 0.0192  (.0725) |
| Female | 0.0173  (.0332) | 0.0493  (.0447) |  |
| Std Height *Female | 0.0058  (.0316) | 0.0253  (.0417) | 0.1507  (.0957) |
| Std Height PGS |  | -0.0099  (.0332) |  |
| Std Education PGS |  | 0.1256  (.0326) |  |
|  |  |  |  |
| R^2^ | 0.002 | 0.021 | 0.010 |
| N | 4, 160 | 2,498 | 2,386 |

*Notes:* Twins fixed effect regressions control for birthweight. Cognition is standardized to have a mean of 0 and standard deviation of 1. Standard errors clustered at the twin pair level. ***p<0.001 **p<0.05 p<0.10

The within R^2^ from twins fixed effect regressions is reported in all columns. Std: Standardized.

**Table S3: Height-Cognition Associations at Age 12 and Age 16, Full Analytical Samples**

| **Sample** | **DZ Twin Pairs** | **DZ Twin Pairs** | **Genotyped DZ Twin Pairs** | **Genotyped DZ Twin Pairs** | **Genotyped DZ Twin**  **Pairs** | **MZ Twin Pairs** | **MZ Twin Pairs** |
| --- | --- | --- | --- | --- | --- | --- | --- |
| **Method** | **OLS** | **FE** | **OLS** | **FE** | **FE** | **OLS** | **FE** |
|  | **(1)** | **(2)** | **(3)** | **(4)** | **(5)** | **(6)** | **(7)** |
| **Panel A: Age 12** | | | | | | | |
| Std Height | 0.0312*  (.0174) | 0.0428*  (.0246) | 0.0178  (.0210) | 0.0559*  (.0301) | 0.0684**  (.0319) | 0.0085  (.0249) | 0.0570  (.0453) |
| Std Height PGS |  |  |  |  | -0.0487  (.0372) |  |  |
| Std Education PGS |  |  |  |  | 0.1254***  (.0343) |  |  |
|  |  |  |  |  |  |  |  |
| R^2^ | 0.183 | 0.005 | 0.172 | 0.008 | 0.020 | 0.160 | 0.012 |
| N | 3,472 | 3,472 | 2,342 | 2,342 | 2,342 | 2,084 | 2,084 |
| **Panel B: Age 16** | | | | | | | |
| Std Height | 0.0557  (.0243) | 0.0027  (.0354) | 0.0361  (.0306) | 0.0160  (.0453) | 0.0018  (.0488) | 0.0622*  (.0330) | 0.1048*  (.0599) |
| Std Height PGS |  |  |  |  | 0.0392  (.0553) |  |  |
| Std Education PGS |  |  |  |  | 0.1831***  (.0511) |  |  |
|  |  |  |  |  |  |  |  |
| R^2^ | 0.113 | 0.008 | 0.106 | 0.006 | 0.030 | 0.139 | 0.022 |
| N | 1,992 | 1,992 | 1,232 | 1,232 | 1,232 | 1,214 | 1,214 |

*Notes:* OLS regressions control for age, gender, birthweight, family SES, and ethnicity. Cognition is standardized to have

a mean of 0 and standard deviation of 1. Standard errors clustered at the twin pair level. ***p<0.001 **p<0.05 p<0.10

The within R^2^ from twins fixed effect regressions is reported in columns 2, 4,5, and 7. Std: Standardized

**SECTION B: DATA APPENDIX FOR AGE 12 AND AGE 16 MEASURES**

Height was self-reported by twins at 12 and 16. At age 12 and 16 tests were administered online. Two verbal (information and vocabulary subsets from the WISC-III-UK) and two nonverbal (WISC-III-UK Picture Completion and Raven’s Standard and Advanced Progressive Matrices) tests were completed at age 12. At age 16, twins completed web-based adaptations of Raven’s Standard and Advanced Progressive and the Mill-Hill Vocabulary Scale. All test scores were standardized. Cognition is measured as the average of the standardized test scores at each age. Summary statistics for the age 12 and 16 analytical samples used in Table S3 are given in Table S4 below.

**Table S4: Summary Statistics for Age 12 and Age 16 Analytical Samples**

| **Sample** | **DZ Twin Pairs** | **Genotyped DZ Twin Pairs** | **MZ Twin Pairs** |
| --- | --- | --- | --- |
|  | **(1)** | **(2)** | **(3)** |
| **Panel A: Age 12** | | | |
| Height (cm) | 170.66 (9.41) | 170.81 (9.42) | 169.15 (9.23) |
| Cognition | 0.14 (0.98) | 0.15 (0.97) | 0.05 (0.99) |
| Female | 0.54 (0.50) | 0.55 (0.50) | 0.61 (0.49) |
| White | 0.94 (0.23) | 1.00 (0.00) | 0.93 (0.26) |
| Childhood SES | 0.39 (0.98) | 0.37 (0.97) | 0.18 (0.95) |
| Birthweight (grams) | 2580 (529) | 2575 (514) | 2458 (547) |
| Absolute *within-twin pair difference* in |  |  |  |
| Height (cm) | 5.70 (4.81) | 5.73 (4.83) | 2.23 (2.76) |
| Cognition | 0.80 (0.64) | 0.80 (0.61) | 0.58 (0.45) |
| Birthweight (grams) | 330 (282) | 319 (274) | 299 (264) |
| N | 3,472 | 2,342 | 2,084 |
| **Panel B: Age 16** | | | |
| Height (cm) | 170.66 (9.41) | 170.81 (9.42) | 169.15 (9.23) |
| Cognition | 0.14 (0.98) | 0.15 (0.97) | 0.05 (0.99) |
| Female | 0.54 (0.50) | 0.55 (0.50) | 0.61 (0.49) |
| White | 0.94 (0.23) | 1.00 (0.00) | 0.93 (0.26) |
| Childhood SES | 0.39 (0.98) | 0.37 (0.97) | 0.18 (0.95) |
| Birthweight (grams) | 2580 (529) | 2575 (514) | 2458 (547) |
| Absolute *within-twin pair difference* in |  |  |  |
| Height (cm) | 9.06 (7.04) | 9.16 (7.08) | 2.88 (3.53) |
| Cognition | 0.87 (0.69) | 0.85 (0.67) | 0.70 (0.58) |
| Birthweight (grams) | 353 (297) | 342 (285) | 291 (255) |
| N | 1,922 | 1,232 | 1,214 |

*Notes:* Standard deviation in parentheses.
